# First Report of a Foodborne *Salmonella enterica* Serovar Gloucester (4:i:l,w) ST34 Strain Harboring *bla*_CTX–M–__55_ and *qnrS* Genes Located in IS*26*-Mediated Composite Transposon

**DOI:** 10.3389/fmicb.2021.646101

**Published:** 2021-04-20

**Authors:** Lili Li, Rikke Heidemann Olsen, Anhua Song, Jian Xiao, Chong Wang, Hecheng Meng, Lei Shi

**Affiliations:** ^1^Institute of Food Safety and Nutrition, Jinan University, Guangzhou, China; ^2^Department of Veterinary and Animal Sciences, Faculty of Health and Medical Sciences, University of Copenhagen, Copenhagen, Denmark; ^3^Guangzhou Food Inspection Institute, Guangzhou, China; ^4^Shandong New Hope Liuhe Group Ltd., Qingdao, China; ^5^School of Food Science and Engineering, South China University of Technology, Guangzhou, China

**Keywords:** *bla*_CTX–M–__55_, *qnrS1*, *Salmonella* Gloucester, ready-to-eat, IS*26*

## Abstract

Extended-spectrum β-lactamases (ESBLs) production and (fluoro)quinolone (FQ) resistance among *Salmonella* pose a public health threat. The objective of this study was the phenotypic and genotypic characterization of an ESBL-producing and nalidixic acid-resistant *Salmonella enterica* serovar Gloucester isolate (serotype 4:i:l,w) of sequence type 34 (ST34) from ready-to-eat (RTE) meat products in China. Whole-genome short and long read sequencing (HiSeq and MinION) results showed that it contained *bla*_CTX–M–__55_, *qnrS1*, and *tetB* genes, with *bla*_CTX–M–__55_ and *qnrS1* located in chromosomal IS*26*-mediated composite transposon (IS*26*–*qnrS1*–IS*3*–Tn*3*–*orf*–*bla*_CTX–M–__55_–IS*Ecp1*–IS*26*). The same genetic structure was found in the chromosome of *S. enterica* subsp. *enterica* serovar Typhimurium strain and in several plasmids of *Escherichia coli*, indicating that the IS*26*-mediated composite transposon in the chromosome of *S.* Gloucester may originate from plasmids of *E. coli* and possess the ability to disseminate to *Salmonella* and other bacterial species. Besides, the structural unit *qnrS1*–IS*3*–Tn*3*–*orf*–*bla*_CTX–M–__55_ was also observed to be linked with IS*Kpn19* in both the chromosomes and plasmids of various bacteria species, highlighting the contribution of the insertion sequences (IS*26* and IS*Kpn19*) to the co-dissemination of *bla*_CTX–M–__55_ and *qnrS1*. To our knowledge, this is the first description of chromosomal *bla*_CTX–M–__55_ and *qnrS* in *S.* Gloucester from RTE meat products. Our work expands the host range and provides additional evidence of the co-transfer of *bla*_CTX–M–__55_ and *qnrS1* among different species of *Salmonella* through the food chain.

## Introduction

*Salmonella enterica* is a leading cause of global bacterial foodborne gastroenteritis (Kirk et al., 2015; Lokken et al., 2016). Severely invasive salmonellosis is recommended to be treated with (fluoro)quinolones (FQs) and extended-spectrum cephalosporin (ESC) antimicrobials. Over the past two decades, *S. enterica* strains that are resistant to ESCs and FQs have emerged among humans, animals, and animal products, which is a worldwide public health concern (Wong et al., 2013; Brown et al., 2018; Lu et al., 2019; Zhang et al., 2019).

Cephalosporin resistance is mediated predominantly by extended-spectrum β-lactamases (ESBLs), AmpC β-lactamases, and carbapenemase (Arlet et al., 2006). These β-lactamases confer resistance to a wide range of β-lactam antibiotics, including penicillins, cephalosporins, and carbapenems. Besides, they are typically associated with multiple antibiotic resistance, such as quinolones, aminoglycosides, chloramphenicol, tetracycline, and trimethoprim–sulfamethoxazole and can transfer together among different bacteria species *via* mobile genetic elements (MGEs), leaving few therapeutic choices (Thomson, 2010; Iwamoto et al., 2017; Qiao et al., 2017; Nadimpalli et al., 2019).

Reduced susceptibility to FQs is associated with chromosomal mutations and the acquisition of antibiotic resistance genes, such as efflux pumps and plasmid-mediated quinolone resistance (PMQR) genes [*qnr*, *aac(6′)-Ib-cr*, *oqxAB*, and *qepA* genes] (Cuypers et al., 2018). PMQR genes confer only a low-level resistance to quinolones. However, they would provide a favorable background for higher resistance to occur (Hooper and Jacoby, 2015). Moreover, the ability to be spread by horizontal gene transfer constitutes a serious concern that should be addressed (Poirel et al., 2012).

Food-producing animals are considered to be the primary reservoir of ESBL-producing and quinolone-resistant *Salmonella* (Nadimpalli et al., 2019; Zhang et al., 2019). Contaminated raw or undercooked meat products are primary vehicles for *Salmonella* transmission to humans (Fearnley et al., 2011; Qiao et al., 2017). ESBL-producing and quinolone-resistant *Salmonella* strains have been detected in various *Salmonella* serotypes from clinical settings and raw meat samples (Wu et al., 2015; Qiao et al., 2017; Zhang et al., 2019). However, to our knowledge, this has not been reported in *S. enterica* serovar Gloucester from ready-to-eat (RTE) food products. Here, we characterize the first ESBL-producing and quinolone-resistant *S.* Gloucester strain from an RTE duck product carrying *bla*_CTX–M–__55_ and *qnrS* genes, isolated during routine surveillance in Guangdong Province, China.

## Materials and Methods

### Strain Isolation and Identification

During our routine surveillance of foodborne pathogens on various food products, a *Salmonella* isolate (named GSJ/2017-Sal.-014, hereafter 17Sal014) was recovered from a roasted duck product in Guangzhou, southern China, in 2017. The isolate was first identified by biochemical confirmation using API 20E test identification test strips (bioMérieux, France) and further by 16S ribosomal RNA (rRNA) gene sequencing using the universal primers 27F (5′-AGAGTTTGATCCTG GCTCAG-3′) and 1492R (5′-GGCTACCTTGTTACGACTT-3′). The serotype was determined by the slide agglutination test using *Salmonella* antisera (SSI Diagnostica, Denmark) according to the Kauffmann–White scheme.

The strain was routinely grown in Luria–Bertani (LB; Guangdong Huankai Microbial Sci. & Tech., Guangzhou, China) broth or agar plates at 37°C for 12–24 h. *Escherichia coli* J53 was cultured in LB broth or agar plates with 150 μg/ml sodium azide (Sigma–Aldrich, St. Louis, MO, United States) and incubated at 37°C for 12–24 h.

### Antibiotic Susceptibility Testing

The susceptibility of 17Sal014 to a panel of antimicrobial drugs (Hangzhou Microbial Reagent Co., Ltd., China)—including ciprofloxacin, cephalosporin II (cefuroxime), cephalosporin III (cefotaxime and ceftazidime), cephalosporin IV (cefepime), tetracycline, doxycycline, cefazolin, gentamicin, trimethoprim, tigecycline, chloramphenicol, fosfomycin, tobramycin, amikacin, netilmicin, piperacillin, ertapenem, imipenem, meropenem, cefoxitin, ampicillin–sulbactam sodium, aztreonam and ampicillin, and amoxicillin–clavulanic acid—was determined by disk diffusion antibiotic susceptibility testing (CLSI, 2016). The minimal inhibitory concentrations (MICs) of 17Sal014 to polymyxin B, cefotaxime, ciprofloxacin, and nalidixic acid (Sigma–Aldrich, St. Louis, MO, United States) were determined by broth microdilution (CLSI, 2016). The production of ESBL was confirmed by the disk diffusion clavulanate inhibition test using ceftazidime and cefotaxime. Reference strain *E. coli* ATCC 25922 served as a quality control.

### Whole-Genome Sequencing and Annotation

The genomic DNA of isolate 17Sal014 was extracted using a commercial DNA extraction kit (Magen, Guangzhou, China) following the manufacturer’s recommendations. The whole genome of the isolate was sequenced on Illumina HiSeq X Ten with 150-bp paired-end reads (MajorBio Co., Shanghai, China) and MinION (Oxford Nanopore, Oxford, United Kingdom). For the MinION platform, the library was prepared using the ONT 1D ligation sequencing kit (SQK-LSK109) with the native barcoding expansion kit (EXP-NBD104). The genome was assembled using a combination of short and long reads by SPAdes (Bankevich et al., 2012) and the Unicycler hybrid assembler (Wick et al., 2017) and annotated by Prokka (Seemann, 2014).

Clonal analysis was assessed by MLST 2.0^1^. PlasmidFinder v2.1 (Carattoli and Hasman, 2020) was used to identify plasmid replicon types. The presence of acquired antibiotic resistance genes and mutations in the quinolone resistance-determining regions (QRDRs) (*gyrA*, *gyrB*, *parC*, and *parE*) was assessed by ResFinder (Zankari et al., 2012) and further determined by BLASTn^2^.

### Conjugation

IS*26* plays a critical role in the dissemination of antibiotic resistance genes in Gram-negative bacteria (Harmer et al., 2014; Harmer and Hall, 2015, 2016). To reveal the transferability of the chromosomally located *bla*_CTX–M–__55_ and *qnrS1*, which is possibly mediated by an IS*26*-flanked composite transposon, a conjugation experiment was conducted by solid mating on a filter (Whatman, Maidstone, United Kingdom) using sodium azide-resistant *E. coli* J53 as a recipient and a selection of transconjugants on LB agar containing 150 μg/ml sodium azide and 4, 8, and 16 μg/ml cefotaxime, respectively. The transfer of plasmid to the transconjugants and possible monomeric circular intermediates of the IS*26* in the transconjugants was confirmed by PCR with a set of specific primers (Supplementary Table 1).

### Nucleotide Sequence Accession Number

The assembly genome sequence of *S.* Gloucester 17Sal014 was deposited in the NCBI database under the accession number SAMN14178317. The Whole Genome Shotgun project has been deposited at DDBJ/ENA/GenBank under the accession number VFRK00000000. The 8,993-bp composite transposon containing *bla*_CTX–M–__55_ and *qnrS1* was deposited in GenBank under the accession number MN619286.1.

## Results

### Identification of *Salmonella* and Antibiotic Susceptibility

The isolate was confirmed as *S. enterica* serovar Gloucester, serotype 4:i:l,w, by biochemical confirmation, 16S rRNA gene sequencing, and serotyping.

The disk diffusion antibiotic susceptibility testing showed that the isolate was resistant to tetracycline, doxycycline, cefazolin, aztreonam, ampicillin, amoxicillin clavulanic acid, and cephalosporins II (cefuroxime) and III (cefotaxime and ceftazidime), intermediate resistant to cephalosporin IV (cefepime), and produces ESBL. The isolate exhibited MIC values of ciprofloxacin, nalidixic acid, and cefotaxime of 0.25, 64, and 128 mg/L, respectively.

### General Features of the *S.* Gloucester 17Sal014 Genome

The complete genome sequence of *S.* Gloucester 17Sal014 contained a circular 4,986,395-bp chromosome with a G + C content of 52.2%. There were 5,089 predicted genes and 4,960 predicted coding sequences (CDs) in the whole genome sequence, including 85 RNA genes consisting of 80 transfer RNAs (tRNAs) and four rRNAs. Multilocus sequence typing analysis showed that 17Sal014 belongs to sequence type 34 (ST34).

Acquired resistance genes against tetracycline (*tetB*), quinolones (*qnrS1*), and cephalosporins (*bla*_CTX–M–__55_) were identified. In addition, four more substitutions in *parC* (T255S, A395S, S469A, and A620T) were detected, which were not identified before.

**FIGURE 1:**
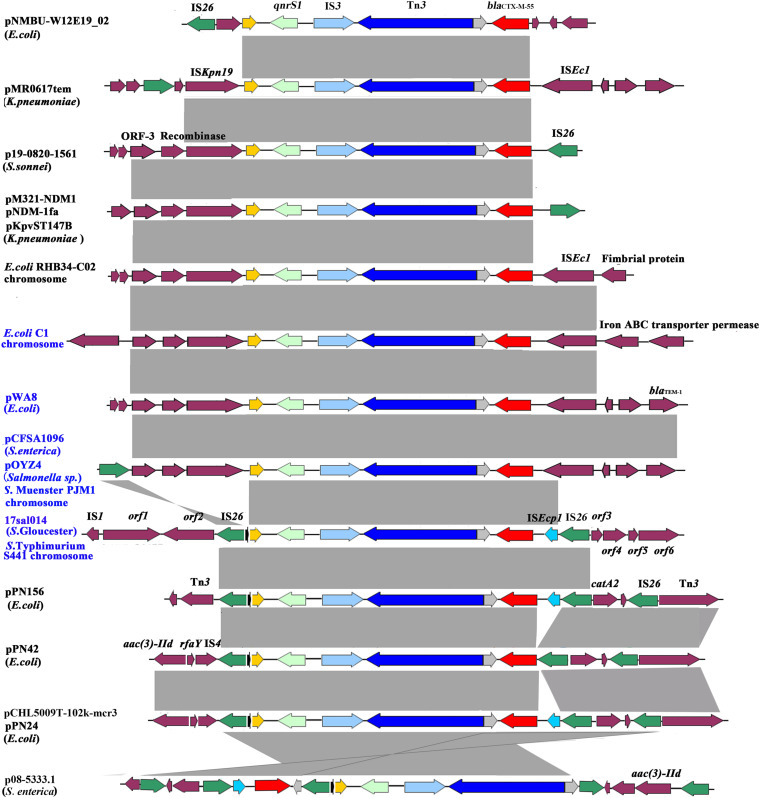
Genetic environment of the *bla*_CTX–M–__55_ and *qnrS1* genes from the *Salmonella* Gloucester isolate 17Sal014 and bacterial species from different regions. The *arrows* indicate open reading frames. *Light gray shading* denotes homology regions. The *orf1*–*orf6* genes encode DNA cytosine methyltransferase, type II site-specific deoxyribonuclease, hypothetical protein, 3′–5′ exoribonuclease, DUF4224 domain-containing protein, and tyrosine-type recombinase/integrase, respectively. Strains from China are in *blue* font.

The *bla*_CTX–M–__55_ and *qnrS1* genes were located on chromosome within 8,993 bp composite transposon, IS*26*–*qnrS1*–IS*3*–Tn*3*–*orf*–*bla*_CTX–M–__55_–IS*Ecp1*–IS*26*, and flanked by genes encoding DNA cytosine methyltransferase (*orf1*), type II site-specific deoxyribonuclease (*orf2*), hypothetical protein (*orf3*), 3′–5′ exoribonuclease (*orf4*), DUF4224 domain-containing protein (*orf5*), and tyrosine-type recombinase/integrase (*orf6*). *bla*_CTX–M–__55_ was linked with incomplete IS*Ecp1*. The 8,993-bp genetic structure was identical to the corresponding region on the chromosome of *Salmonella* Typhimurium strain S441 isolated from a human stool sample in Hangzhou, China (GenBank accession no. CP061122.1) and with 99% identity (with 100% query coverage) to pPN156 (GenBank accession number MT449721.1) of an *E. coli* strain from human intestine and pPN24 (GenBank accession number MT449722.1) of an *E. coli* strain from duck feces in Thailand, as well as pCHL5009T-102k-mcr3 (GenBank accession number CP032937.1) of an *E. coli* strain from urine samples in New Zealand. The plasmid p08-5333.1 of *S. enterica* subsp. *enterica* strain (GenBank accession number CP039562.1) shared a similar genetic environment, but differed in *orf-bla*_CTX–M–__55_–IS*Ecp1*–IS*26* insertion site and orientation. The pPN42 of the *E. coli* strain (GenBank accession number MT449720.1) also contained a similar region, but lacked IS*Ecp1*. Besides, the structural unit *qnrS1*–IS*3*–Tn*3*–*orf*–*bla*_CTX–M–__55_ was observed in various bacterial species, such as the chromosome of *E. coli* strains and plasmids in *E. coli*, *Klebsiella pneumoniae*, *Salmonella* Typhi, and *S. enterica* (Figure 1 and Supplementary Table 2). Of note is that the unit was identified in either the chromosome or on plasmids in four *Salmonella* and two *E. coli* strains of animal, chicken meat, food, and human sources in China. In these isolates from China and various species from other countries, the unit *qnrS1*–IS*3*–Tn*3*–*orf*–*bla*_CTX–M–__55_ was linked with IS*Kpn19* and/or IS*26* (Table 1 and Figure 1). These findings indicate that the structural unit *qnrS1*–IS*3*–Tn*3*–*orf*–*bla*_CTX–M–__55_ was transferred among different bacterial species with the help of the insertion sequences (IS*26* and IS*Kpn19*). The transfer of *bla*_CTX–M–__55_ and *qnrS1* to *E. coli* was not detected under laboratory experimental conditions in this study. However, it is possible that the IS*26*-mediated composite transposon maintains the transfer capacity under adverse environments.

**TABLE 1:** Characteristics of *qnrS1*–IS*3*–Tn*3*–*orf*–*bla*_CTX–M–__55_ harboring isolates in China.

Strains	*bla*_CTX–M–__55_ location	Source	Year	Resistance characterization				Accession number
				MIC_*CTX*_	Cephalosporins	MIC_*CIP*_	Quinolone	
*E. coli* WA8	Plasmid PWA8	Animal	–	–	*bla*_CTX–M–__55_, *bla*_*TEM–*__1_	–	*qnrS*	MG773378.1
*E. coli* C1	Chromosome	Cow	2014	–	*bla*_CTX–M–__55_, *bla*_*EC*_	–	*qnrS*	CP010116.1
*S. enterica* strain CFSA1096	Plasmid pCFSA1096	Food	2015	–	*bla*_CTX–M–__55_, *bla*_*TEM–*__1_, *bla*_*LAP–*__2_	–	*qnrS*	CP033347.2
*S.* Muenster strain PJM1	Chromosome	Chicken meat	2017	128	*bla*_CTX–M–__55_, *bla*_*TEM–*__1_, *bla*_*OXA–*__1_	64	*qnrS*	CP045038.1
*S.* Typhimurium strain S441	Chromosome	Human stool	2017	–	*bla*_CTX–M–__55_, *bla*_*TEM–*__1_	–	*qnrS*	CP061122.1
*Salmonella* sp. strain OYZ4	Plasmid pOYZ4	Duck	2017	128	*bla*_CTX–M–__55_, *bla*_*TEM–*__1_, *bla*_*OXA–*__1_	4	*qnrS*	MN539018.1
*S.* Gloucester 17Sal014	Chromosome	RTE duck	2017	128	*bla*_CTX–M–__55_	0.25	*qnrS*	SAMN14178317

*RTE, ready to eat; CTX, cefotaxime; CIP, ciprofloxacin.*

## Discussion

ESBL-producing and quinolone-resistant strains of *Salmonella*, which constitute a great public health concern, have been increasingly reported throughout the world (Castellanos et al., 2018; Nadimpalli et al., 2019; Zhang et al., 2019). Previous studies have reported the presence of ESBL-producing and quinolone-resistant *Salmonella* isolates from humans (Wong et al., 2014), healthy pigs and chickens (de Jong et al., 2014), pig feces and tonsils at slaughter (Van Damme et al., 2017), and retail pork and chicken meat (Qiao et al., 2017; Brown et al., 2018; Nadimpalli et al., 2019). These data highlight that the food of animals is an important reservoir of ESBL-producing and quinolone-resistant *Salmonella*. However, ESBL-producing and quinolone-resistant *Salmonella* strains have only rarely been reported in RTE meat products. In this study, we identified an *S.* Gloucester strain isolated from an RTE duck product in China harboring the ESBL-encoding *bla*_CTX–M–__55_ gene conferring ESC resistance and *qnrS1* encoding quinolone resistance.

*Salmonella* Gloucester is a rarely reported *S. enterica* serovar, only sporadically found in human and retail chicken meat samples (Hoque et al., 1994; Azemi et al., 2013; Tîrziu et al., 2020). Of the reported *S.* Gloucester isolates from clinical cases, resistance to ampicillin, chloramphenicol, trimethoprim–sulfamethoxazole, and ceftriaxone was reported (Rahman et al., 2001). However, no sequence information of the genetic determinants of these strains is publicly available. There are only two whole-genome sequences of *S.* Gloucester strains from clinical samples in the United Kingdom deposited in NCBI and EnteroBase (accession number SRR7842640). The two isolates contained no acquired antibiotic resistance gene by searching against the ResFinder database. In contrast, the *S.* Gloucester 17sal1004 strain isolated in this study showed a multiple antibiotic resistance phenotype and contained multiple resistance genes. Of note is that this strain exhibited high-level resistance to third-generation cephalosporins, produced ESBL, and are resistant to nalidixic acid, which represents a public health concern due to the possibility of dissemination through the food chain. Improved sanitary practices are important to help control the transmission of the ESBL-producing *S.* Gloucester to humans through the food chain.

*Salmonella* resistance to ESCs is reported to be associated with cross-resistance to FQs (Lu et al., 2019; Zhang et al., 2019). In the current study, *S.* Gloucester strain was susceptible to ciprofloxacin, but resistant to nalidixic acid, and it harbored PMQR genes *qnrS1* as well as contained resistance-associated mutations in *parC*. The existence of *qnrS* and the mutations in *parC* provide strains with a selective advantage under quinolone exposure and can accelerate the development of chromosome-mediated quinolone resistance (Jacoby et al., 2014; Rodríguez-Martínez et al., 2016).

CTX-M-55 is an emerging ESBL type with enhanced cephalosporin-hydrolyzing activity (He et al., 2015). Since initially identified in *E. coli* of human origin in the United States and China in 2011, *bla*_CTX–M–__55_ has been increasingly identified in *E. coli*, *K. pneumonia*, and in several *Salmonella* serovars from humans, animals, and food of animals, as well as in the environment (Shi et al., 2008; Lupo et al., 2018; Nadimpalli et al., 2019; Dong et al., 2020; Li et al., 2021). Recent studies in China (Zhang et al., 2019), Cambodia (Nadimpalli et al., 2019), and Thailand (Luk-In et al., 2018) showed the high frequency of the CTX-M-55 type in different *Salmonella* serovars among food animals and retail meats, which suggests that CTX-M-55 transferred rapidly cross *Salmonella* species and could be stably persistent in *Salmonella* in food animals.

The *bla*_CTX–M–__55_ gene is reported to be mostly located on epidemic self-mobilizable plasmids, such as IncF, IncI1 and IncHI2, and IncA/C2 (Wang et al., 2018). However, a recent study from China demonstrated a high frequency of chromosomal copies of *bla*_CTX–M–__55_ in CTX-M-55-producing *Salmonella* strains and the transferability of the chromosomal *bla*_CTX–M–__55_ to *E. coli* by conjugation (Zhang et al., 2019). The *bla*_CTX–M–__55_ gene was also found in the chromosome of *S.* Gloucester 17Sal014 in the present study. Moreover, the *bla*_CTX–M–__55_ gene was identified to be located in an IS*26-*mediated composite transposon, IS*26*–*qnrS1*–IS*3*–Tn*3*–*orf*–*bla*_CTX–M–__55_–IS*Ecp1*–IS*26*, with the structural unit *qnrS1*–IS*3*–Tn*3*–*orf*–*bla*_CTX–M–__55_–IS*Ecp1* similar to the corresponding region in the chromosome of *S*. Muenster strain PJM1 (GenBank accession number CP045038.1) and the plasmid of a *Salmonella* sp. isolate (GenBank accession number MN539018.1), as described in Zhang et al. (2019). Similar structure units linked with IS*Kpn19*, instead of IS*26*, were seen in the chromosomes and plasmids of different bacterial species from China and other countries, suggesting that both IS*26* and IS*Kpn19* contributed to the transfer of the *qnrS1*- and *bla*_CTX–M–__55_-carrying unit to chromosomes and plasmids among different bacterial species.

The *bla*_CTX–M–__55_ gene was reported to be often encoded in typical IS*Ecp1*–*bla*_CTX–M–__55_–ORF*477* format on various plasmids, such as pSTH21, pHN1122-1, and p1081-CTXM (Lv et al., 2013; Qu et al., 2014; Wong et al., 2015; Li et al., 2021). However, we found that IS*Ecp1* was incomplete and inserted with IS*26*, which was also inserted on the other end of the structural unit *qnrS1*–IS*3*–Tn*3*–*orf*–*bla*_CTX–M–__55_–IS*Ecp1*. This configuration might have resulted from the insertion of the IS*Ecp1*–*bla*_CTX–M–__55_ transposition unit into a plasmid backbone and capture of the adjacent fragment, as well as IS*26* in a subsequent transposition event (Lv et al., 2013; Qu et al., 2014; Wong et al., 2015). The unit, combined with the bounded two copies of IS*26*, constitutes a composite transposon which was able to transfer by forming a circular molecule (Harmer et al., 2014; Harmer and Hall, 2015, 2016). In particular, the identical IS*26*-mediated composite transposon was observed to exist mainly on plasmids of *E. coli*. It is likely that the IS*26*-mediated composite transposon in the chromosome of *S.* Gloucester in this study originated from the plasmid of *E. coli* and possesses the ability to disseminate to *Salmonella* and other bacterial species. The IS*26*-mediated composite transposon IS*26*–*qnrS1*–IS*3*–Tn*3*–*orf*–*bla*_CTX–M–__55_–IS*Ecp1*–IS*26* was found in *Salmonella* and *E. coli* strains of animal, chicken meat, food, RTE food, and human sources in China, suggesting that it has spread along the food chain, which may contribute to the development of antibiotic resistance to cephalosporin and fluoroquinolones.

## Conclusion

To the best of our knowledge, we describe for the first time an ESBL-producing and quinolone-resistant *S.* Gloucester (4:i:l,w) ST34 strain from an RTE duck product co-harboring the chromosomally located *bla*_CTX–M–__55_ and *qnrS1*. These strains represent potential clinical and food safety issues since they may transmit to humans through the food chain and may lead to a reduced susceptibility of *Salmonella* to critical antibiotics, cephalosporins and fluoroquinolones, that are front-line drugs of choice for treating severe *Salmonella* infections. The structural unit *qnrS1*–IS*3*–Tn*3*–*orf*–*bla*_CTX–M–__55_, combined with IS*26* and IS*Kpn19*, contributed to the co-transfer of the *bla*_CTX–M–__55_ and *qnrS* genes into the chromosomes or plasmids of different bacterial species, which may accelerate the development and dissemination of isolates co-resistant to cephalosporins and fluoroquinolones. This warrants continuous investigation of the coexistence and the co-transfer mechanisms of *bla*_CTX–M–__55_ and *qnrS* among different bacterial species from various sources in China.

## Data Availability Statement

The datasets presented in this study can be found in online repositories. The names of the repository/repositories and accession number(s) can be found in the article/Supplementary Material.

## Author Contributions

AS and JX conceptualized and designed the study. LL and HM performed the experiment and wrote the manuscript. CW was involved in the generation of short and long read sequencing of the genomes. LS and RO assisted in revising the manuscript. All authors contributed to the article and approved the submitted version.

## Conflict of Interest

CW was employed by the company Shandong New Hope Liuhe Group Ltd., Qingdao. The remaining authors declare that the research was conducted in the absence of any commercial or financial relationships that could be construed as a potential conflict of interest.

## Footnotes

1
http://mlst.warwick.ac.uk/mlst/dbs/Senterica

2
http://blast.ncbi.nlm.nih.gov/Blast.cgi
