# Corrigendum: First Report of a Foodborne *Salmonella enterica* Serovar Gloucester (4:i:l,w) ST34 Strain Harboring *bla*_CTX–M−55_ and *qnrS* Genes Located in IS*26*-Mediated Composite Transposon

**DOI:** 10.3389/fmicb.2021.792791

**Published:** 2021-11-02

**Authors:** Lili Li, Rikke Heidemann Olsen, Anhua Song, Jian Xiao, Chong Wang, Hecheng Meng, Lei Shi

**Affiliations:** ^1^Institute of Food Safety and Nutrition, Jinan University, Guangzhou, China; ^2^Department of Veterinary and Animal Sciences, Faculty of Health and Medical Sciences, University of Copenhagen, Copenhagen, Denmark; ^3^Guangzhou Food Inspection Institute, Guangzhou, China; ^4^Shandong New Hope Liuhe Group Ltd., Qingdao, China; ^5^School of Food Science and Engineering, South China University of Technology, Guangzhou, China

**Keywords:** *bla*_CTX–M–55_, *qnrS1*, *Salmonella* Gloucester, ready-to-eat, *IS26*

In the original article, there was an error. We wrote the wrong name for the supplier, instead of “Statens Serum Institute” it should be “SSI Diagnostica.”

A correction has been made to **Materials and Methods, Strain Isolation and Identification**, paragraph 1:

“During our routine surveillance of foodborne pathogens on various food products, a *Salmonella* isolate (named GSJ/2017-Sal.-014, hereafter 17Sal014) was recovered from a roasted duck product in Guangzhou, southern China, in 2017. The isolate was first identified by biochemical confirmation using API 20E test identification test strips (bioMérieux, France) and further by 16S ribosomal RNA (rRNA) gene sequencing using the universal primers 27F (5′-AGAGTTTGATCCTG GCTCAG-3′) and 1492R (5′-GGCTACCTTGTTACGACTT-3′). The serotype was determined by the slide agglutination test using *Salmonella* antisera (SSI Diagnostica, Denmark) according to the Kauffmann–White scheme.”

The authors apologize for this error and state that this does not change the scientific conclusions of the article in any way. The original article has been updated.

## Publisher's Note

All claims expressed in this article are solely those of the authors and do not necessarily represent those of their affiliated organizations, or those of the publisher, the editors and the reviewers. Any product that may be evaluated in this article, or claim that may be made by its manufacturer, is not guaranteed or endorsed by the publisher.

