# Supplementary material for: First Report of a Foodborne Salmonella enterica Serovar Gloucester (4:i:l,w) ST34 Strain Harboring blaCTX–M–55 and qnrS Genes Located in IS26-Mediated Composite Transposon
Source: Front Microbiol. 2021 Apr 20;12:646101. doi: 10.3389/fmicb.2021.646101 (PMC8093823; doi:10.3389/fmicb.2021.646101)
Supplement: Supplementary file 1 [file Data_Sheet_1.docx]

**Supplementary data**

**Supplementary Table S1.** Primers used for PCR in this study.

| Primer | Sequence (5′-3′) | Target gene | Size(bp) | Reference |
| --- | --- | --- | --- | --- |
| bla_CTX-M-55_-F | AAGCACGTCAATGGGACGAT | *bla*CTX-M-55 | 478 | this study |
| bla_CTX-M-55_-R | CCTTAGGTTGAGGCTGGGTG |  |  | this study |
| IS26-resolvase-F | GTGGCAGATCCCACGATTCA | IS26-resolvase | 455 | this study |
| IS26-resolvase-R | CGGCTTCATTCGCCCAAAAT |  |  | this study |
| qnrS1-F | TAGAGTTCCGTGCGTGTGAT | *qnrS1* | 293 | this study |
| qnrS1-R | GAGTTCGGCGTGGCATAAAT |  |  | this study |

**Supplementary Table S2.** Species contains qnrS1-IS3-Tn3-orf-bla_CTX-M-55_ (with 100% of coverage and significant identity) according to Blastn alignment using NCBI GenBank database.

| **Species** | **Query Cover** | **Per. Ident** | **Location** | **Accession** |
| --- | --- | --- | --- | --- |
| *Enterobacteriaceae bacterium* | 100% | 99.99% | Plasmid | MN657248.1 |
| *Escherichia albertii* | 100% | 99.97% | Plasmid | CP070297.1 |
| *Escherichia coli* | 100% | 100.00% | Plasmid | CP032937.1 |
| *Escherichia coli* | 100% | 100.00% | Plasmid | MT449721.1 |
| *Escherichia coli* | 100% | 100.00% | Plasmid | MT449720.1 |
| *Escherichia coli* | 100% | 99.99% | Plasmid | MT449722.1 |
| *Escherichia coli* | 100% | 100.00% | Plasmid | AP023198.1 |
| *Escherichia coli* | 100% | 100.00% | Plasmid | AP023191.1 |
| *Escherichia coli* | 100% | 100.00% | Plasmid | LC511658.1 |
| *Escherichia coli* | 100% | 99.99% | Plasmid | AP023232.1 |
| *Escherichia coli* | 100% | 99.99% | Plasmid | CP042974.1 |
| *Escherichia coli* | 100% | 99.99% | Plasmid | CP042900.1 |
| *Escherichia coli* | 100% | 99.99% | Plasmid | CP042902.1 |
| *Escherichia coli* | 100% | 99.99% | Plasmid | CP042872.1 |
| *Escherichia coli* | 100% | 99.99% | Plasmid | MG773378.1 |
| *Escherichia coli* | 100% | 99.99% | Plasmid | CP026200.1 |
| *Escherichia coli* | 100% | 99.99% | Plasmid | MF510423.1 |
| *Escherichia coli* | 100% | 99.99% | Plasmid | CP021881.1 |
| *Escherichia coli* | 100% | 99.99% | Plasmid | CP021681.1 |
| *Escherichia coli* | 100% | 99.99% | Plasmid | KY051550.1 |
| *Escherichia coli* | 100% | 99.99% | Plasmid | CP018207.1 |
| *Escherichia coli* | 100% | 99.99% | Plasmid | LC056159.1 |
| *Escherichia coli* | 100% | 99.99% | Plasmid | LC056403.1 |
| *Escherichia coli* | 100% | 99.99% | Plasmid | KM023153.1 |
| *Escherichia coli* | 100% | 99.99% | Plasmid | LR999867.1 |
| *Escherichia coli* | 100% | 99.99% | Plasmid | LR999865.1 |
| *Escherichia coli* | 100% | 99.99% | Chromosome | CP068823.1 |
| *Escherichia coli* | 100% | 99.99% | Plasmid | LR882051.1 |
| *Escherichia coli* | 100% | 99.99% | Chromosome | CP050212.1 |
| *Escherichia coli* | 100% | 99.99% | Chromosome | CP050210.1 |
| *Escherichia coli* | 100% | 99.99% | Chromosome | CP050209.1 |
| *Escherichia coli* | 100% | 99.99% | Chromosome | CP050208.1 |
| *Escherichia coli* | 100% | 99.99% | Chromosome | CP050203.1 |
| *Escherichia coli* | 100% | 99.99% | Chromosome | CP050196.1 |
| *Escherichia coli* | 100% | 99.99% | Chromosome | CP057185.1 |
| *Escherichia coli* | 100% | 99.99% | Chromosome | CP057072.1 |
| *Escherichia coli* | 100% | 99.99% | Plasmid | CP056606.1 |
| *Escherichia coli* | 100% | 99.99% | Plasmid | AP022263.1 |
| *Escherichia coli* | 100% | 99.97% | Plasmid | LR595881.1 |
| *Escherichia coli* | 100% | 99.97% | Chromosome | CP070232.1 |
| *Escherichia coli* | 100% | 99.97% | Plasmid | CP069974.1 |
| *Escherichia coli* | 100% | 99.96% | Plasmid | AP023221.1 |
| *Escherichia coli* | 100% | 99.94% | Plasmid | CP059930.1 |
| *Escherichia coli* | 100% | 99.88% | Plasmid | CP044306.1 |
| *Escherichia coli* | 100% | 99.99% | Plasmid | AP022178.1 |
| *Escherichia coli* O10:H32 | 100% | 99.97% | Plasmid | CP042886.1 |
| *Escherichia coli* O169:H41 | 100% | 99.99% | Plasmid | CP024226.1 |
| *Klebsiella pneumoniae* | 100% | 100.00% | Plasmid | CP024461.1 |
| *Klebsiella pneumoniae* | 100% | 99.99% | Plasmid | CP052151.1 |
| *Klebsiella pneumoniae* | 100% | 99.99% | Plasmid | CP044029.1 |
| *Klebsiella pneumoniae* | 100% | 99.99% | Plasmid | AP018834.1 |
| *Klebsiella pneumoniae* | 100% | 99.99% | Plasmid | CP026158.1 |
| *Klebsiella pneumoniae* | 100% | 99.99% | Plasmid | CP028178.1 |
| *Klebsiella pneumoniae* | 100% | 99.99% | Plasmid | CP025517.1 |
| *Klebsiella pneumoniae* | 100% | 99.99% | Plasmid | CP023918.1 |
| *Klebsiella pneumoniae* | 100% | 99.99% | Plasmid | CP023910.1 |
| *Klebsiella pneumoniae* | 100% | 99.99% | Plasmid | CP021941.1 |
| *Klebsiella pneumoniae* | 100% | 99.99% | Plasmid | CP021947.1 |
| *Klebsiella pneumoniae* | 100% | 99.99% | Plasmid | CP014757.1 |
| *Klebsiella pneumoniae* | 100% | 99.99% | Plasmid | KJ187751.1 |
| *Klebsiella pneumoniae* | 100% | 99.99% | Plasmid | CP009115.1 |
| *Klebsiella pneumoniae* | 100% | 99.99% | Plasmid | AP022554.1 |
| *Klebsiella pneumoniae* | 100% | 99.84% | Plasmid | CP040728.1 |
| *Klebsiella pneumoniae* subsp. *pneumoniae* | 100% | 99.99% | Plasmid | CP034085.1 |
| *Klebsiella pneumoniae* subsp. *pneumoniae* | 100% | 99.94% | Plasmid | CP034201.2 |
| *Salmonella enterica* | 100% | 99.99% | Plasmid | CP046430.1 |
| *Salmonella enterica* subsp. *enterica* | 100% | 100.00% | Plasmid | CP033347.2 |
| *Salmonella enterica* subsp*. enterica* serovar *Agona* | 100% | 99.99% | Plasmid | CP048776.1 |
| *Salmonella enterica* subsp. *enterica* serovar *Goldcoast* | 100% | 99.96% | Plasmid | CP039170.1 |
| *Salmonella enterica* subsp*. enterica* serovar *Goldcoast* | 100% | 99.96% | Plasmid | CP037959.1 |
| *Salmonella enterica* subsp. *enterica* serovar *Goldcoast* | 100% | 99.96% | Plasmid | CP062226.1 |
| *Salmonella enterica* subsp. *enterica* serovar *Goldcoast* | 100% | 99.96% | Plasmid | CP062224.1 |
| *Salmonella enterica* subsp. *enterica* serovar *Kentucky* | 100% | 100.00% | Plasmid | CP039440.1 |
| *Salmonella enterica* subsp. *enterica* serovar *Muenster* | 100% | 99.97% | Chromosome | CP045038.1 |
| *Salmonella enterica* subsp. *enterica* serovar *Newport* | 100% | 100.00% | Plasmid | CP039438.1 |
| *Salmonella enterica* subsp. *enterica* serovar *Newport* | 100% | 100.00% | Chromosome | CP039436.1 |
| *Salmonella enterica* subsp. *enterica* serovar *Typhi* | 100% | 99.99% | Plasmid | CP044008.1 |
| *Salmonella enterica* subsp*. enterica* serovar *Typhi* | 100% | 99.99% | Plasmid | CP040574.1 |
| *Salmonella enterica* subsp*. enterica* serovar *Typhi* | 100% | 99.99% | Plasmid | LT906492.1 |
| *Salmonella enterica* subsp. *enterica* serovar *Typhi* | 100% | 99.99% | Plasmid | LT882487.1 |
| *Salmonella enterica* subsp*. enterica* serovar *Typhimurium* | 100% | 100.00% | Chromosome | CP061122.1 |
| *Salmonella sp.* | 100% | 100.00% | Plasmid | MN539018.1 |
| *Salmonella sp.* | 100% | 100.00% | Plasmid | MN539017.1 |
| *Shigella flexneri* | 100% | 99.99% | Plasmid | CP045523.1 |
| *Shigella sonnei* | 100% | 99.99% | Plasmid | CP049174.1 |
| *Shigella sonnei* | 100% | 99.97% | Plasmid | CP045525.2 |
| *Shigella sonnei* | 100% | 99.97% | Plasmid | CP049186.1 |
| *Shigella sonnei* | 100% | 99.97% | Plasmid | CP049170.1 |
